# Raman Peaks Feature-Based Machine Learning for Raman Spectroscopy Quantification of Inorganic Pollutants

**DOI:** 10.3390/s26144552

**Published:** 2026-07-17

**Authors:** Antonio Nocera, Michela Raimondi, Lorenzo Luciani, Laura Burattini, Laura Falaschetti, Rossana Galassi

**Affiliations:** 1 Dipartimento di Ingegneria dell’Informazione, Università Politecnica delle Marche, 60131 Ancona, Italy; 2 Scuola di Scienze e Tecnologie, Chemistry Division, Università di Camerino, 62032 Camerino, Italy

**Keywords:** Raman spectroscopy, machine learning, quantification, water analysis, nitrate, nitrite, sulphate

## Abstract

This study investigates how Raman peak features influence the accuracy of pollutant concentration quantification in Raman spectroscopy using feature-based machine learning. A Raman peak feature-based approach is applied to data acquired from laboratory Raman equipment for inorganic anion mixtures, with the objective of identifying processing strategies suitable for low-resolution Raman instruments. A dataset comprising nitrate, nitrite, and sulphate dissolved in water at varying concentrations was used to develop a pre-processing pipeline, train and validate models, and perform linear regression using peak- and area-based features extracted from analyte-specific spectral fingerprint regions. Signal downsampling was employed to simulate reduced spectral resolution and evaluate feature robustness under peak broadening conditions. Results indicate that peak-based regression is more sensitive to reduced peak resolution for nitrate and sulphate, whereas area-based regression produces more stable prediction errors across downsampling factors. Conversely, for nitrite, peak-based regression is less affected by downsampling, while area-based regression exhibits degraded performance. Area-based regression achieved mean absolute percentage errors below 7% and 16% for nitrate concentrations above 3833 mg/L and 1916 mg/L, respectively, and generally below 16% for sulphate concentrations above 1916 mg/L. For nitrite, peak-based regression yielded errors within 4%.

## 1. Introduction

Water quality is a major environmental and public health concern, largely due to contamination arising from agricultural and industrial activities [1,2].

Raman spectroscopy has emerged as a promising technique for water analysis, primarily because water induces minimal spectral interference compared to infrared and fluorescence-based methods [3]. It enables both qualitative and quantitative characterization of multiple dissolved species. Qualitative analysis relies on the identification of distinct vibrational fingerprints of different compounds and has shown promising results in applications for the detection of microplastics [4] and inorganic pollutants [5].

In contrast, quantitative analysis is based on Raman peak intensity within specific fingerprint regions. This task is inherently more challenging, as Raman signal intensity is expressed in arbitrary units and must be appropriately processed to yield accurate concentration estimates. Recent advances demonstrate that combining Raman peak processing techniques with machine learning methods can significantly improve quantification performance, thereby extending the capabilities of traditional Raman spectroscopy [6].

Additionally, Raman spectroscopy is non-destructive and requires minimal sample preparation, making it suitable for rapid and *in situ* water quality monitoring [7]. Consequently, a growing trend is the deployment of miniaturized Raman instruments for field applications [8], which, compared to laboratory systems, may suffer from reduced spectral resolution and lower signal quality, even though optimal spectral resolutions are often presented [9].

Poorly resolved Raman peaks pose a significant limitation for both qualitative and quantitative analyses. It leads to broader spectral features, loss of fine details, and increased overlap between characteristic peaks. As a result, compound discrimination becomes more difficult, and quantification accuracy may degrade due to peak distortion, shifts, and overlap.

The impact of Raman peak features on the performance of concentration prediction by machine learning has not been systematically investigated in the context of Raman spectroscopy for water pollutants.

In this work, we propose a method for quantifying dissolved substances in water using Raman spectroscopy, with a specific focus on robustness to loss of peak intensity. Currently, there are no standardized guidelines regarding spectral resolution, and commercial portable spectrometers typically operate at lower resolutions than laboratory systems. This study aims to identify features that enable reliable predictions under conditions of poorly resolved Raman peak features.

To this end, we address the following research questions:

- Q1: How does a reduction in spectral resolution affect the evaluation of already hardly discernable Raman signals?
- Q2: What is the impact of Raman peak features on concentration prediction performance, and which peak features are most robust to *dispersion per pixel* changes?

The experimental setup and the employed dataset are described in Section 2. Section 3 describes the process of development of the pre-processing steps and machine learning pipeline for the predictor of the analytes’ concentrations. Section 4 presents and discusses the results to answer the aforementioned research questions. Eventually, Section 7 concludes the paper.

### Current State of the Art

The effectiveness of Raman spectroscopy is frequently constrained by low signal-to-noise ratios and spectral artifacts such as baseline drift and cosmic ray interference [10,11]. These noise components often render the relationship between signal intensity and analyte concentration non-linear and poorly repeatable, necessitating advanced computational frameworks to transform raw optical indications into robust digital measurements [6,12]. Historically, spectral correction relied on parameter-heavy manual pre-processing, but recent advancements are shifting the paradigm toward integrated, open-source modular packages and deep learning models [10,13]. To address methodological fragmentation, frameworks like RamanSPy and PyFasma provide standardized infrastructures that streamline the entire data life cycle [12,13]. These packages integrate essential pre-processing tools, including despiking, smoothing, and baseline correction, with multivariate statistical techniques such as Principal Component Analysis (PCA) and Partial Least Squares Discriminant Analysis (PLS-DA) [12,13]. By facilitating reproducible pipelines, these tools enable robust assessments [6,12]. The integration of deep learning further accelerates Raman analysis by automating the removal of complex artifacts. The RADAR framework utilizes convolutional neural network (CNN) architectures to decompose raw signals into four distinct components: peaks, baseline, noise, and cosmic rays [10]. This simultaneous correction has demonstrated a 90% reduction in required data acquisition time while preserving signal integrity [10]. However, while CNNs offer significant speed and accuracy, they often function as “black boxes” that lack interpretability and may require extensive synthetic datasets to prevent overfitting [6,11]. For this reason, models such as Peak-Sensitive Elastic-net Logistic Regression (PSE-LR) prioritize interpretability by utilizing regularization terms that identify adjacent wavenumbers as cohesive peaks [11]. PSE-LR is highly sensitive to subtle signal differences, detecting intensity variations as small as 3–5% [11]. Complementary to this, the SSNet framework employs self-supervised learning and Gaussian peak masks to unmix trace targets from unknown matrices [14]. This approach achieves expert-level sensitivity in identifying structurally similar molecules, such as gelsemium phytotoxins in food samples, even when signals are invisible to the naked eye [14]. Recent research has extended these AI-assisted methodologies to environmental monitoring, treating machine learning as a virtual metrology layer for quantifying inorganic pollutants in water [6]. This approach utilizes exhaustive grid searches to identify optimal pre-processing pipelines that minimize sample variance and ensure metrological repeatability [6]. By transitioning from raw sensor feeds to parametric Gaussian fitting, these systems remain stable against noise [6]. For quantifying anions like nitrate, sulfate, and nitrite, simple linear regression models built on extracted peak features have been shown to outperform complex non-linear models, maintaining prediction errors below 10% for concentrations exceeding 1000 mg/L [6]. A fundamental methodological shift can be observed from qualitative spectral interpretation to automated, quantitative, and interpretable digital measurements [6,11]. The combination of lightweight deep learning for artifact removal, peak-sensitive modeling for classification, and standardized open-source software provides a scalable path for deploying Raman sensors in in situ environmental sensing [6,10,13]. These innovations collectively improve specificity in multi-analyte mixtures while reducing the necessity for extensive sample preparation and expert supervision [6,11,14].

## 2. Raman Acquisitions

The experimental setup was previously described in [6]. In this analysis set, the Raman optical spectroscopy acquisitions were carried out using a micro-Raman system Horiba iHS320 equipped with two Olympus microscopes, which encompasses a versatile experimental configuration and the application of different laser sources based on the material being examined: a 532 nm DPSS laser, a 633 nm He-Ne laser, and a 785 nm solid-state laser. After an initial preliminary set of acquisitions with laser configurations, power outputs, and acquisition times, it was chosen to operate with the green 532 nm DPSS laser, by recording 10 scans with an integration time of 5 s for each spectrum (50 s total), repeating 10 times at any given concentration, and with a selected 600 g/mm grating. The system’s use of optical fibers enables operation with laser beam dimensions ranging from 2 μm to 5 μm. The scattered photons were collected in a backscattering geometry and analyzed by the spectrometer (Horiba iHS320) configured in Czerny–Turner geometry, equipped with a Horiba Sincerity CCD7 camera detector. The instrumental setup described herein allows the acquisition of spectral data within the Stokes region, covering a wavenumber range from 70 cm^−1^ to 6700 cm^−1^; however, the specific spectral range of interest selected for our analysis was from 400 to 3900 cm^−1^, as it includes the anticipated Raman shifts of the species under investigation, including the asymmetric stretching peak of water (the solvent), which was used for the normalization process. Further, the spectral pre-processing and feature extraction process was carried out in the 900 to 3200 cm^−1^ range. The analyses were conducted on single-analyte solutions of nitrates, nitrites, and sulphates, as well as various mixture combinations, within the concentration range of 15,000 to 200 mg/L. The aqueous solutions were placed in an open vial to enable optical focus approximately 800 μm below the liquid surface, using a 50× microscope objective magnification. The selected conditions provided an optimal balance between sensitivity, spectral resolution, and total acquisition time per spectrum.

The dataset description, reporting the mixture of analytes and the concentrations, is reported in Table 1.

In particular, three single-analyte datasets are generated for nitrate, nitrite, and sulphate, respectively. Then, solutions with a mixture of two analytes, either nitrate and nitrite, or nitrate and sulphate, are generated. Eventually, mixtures of the three analytes are generated. The single-analyte scenario is used to decide the possible pre-processing and to train the models for regression purposes. The generalization of these models is tested on the mixture scenarios, which are more complex due to the interactions between the different analytes.

## 3. Machine Learning Development

In the following sub-section, the process of development of the machine learning models is explained.

### 3.1. Downsampling

The previously mentioned datasets are processed to reduce the resolution of the signal to check the effect that the change in peak features has on the performance of the same methodology, from the pre-processing algorithms to the machine learning models. The downsampling algorithm employed is a sliding-window average with no overlap, where the window dimension or downsampling factor is chosen according to the wanted reduction in sampling. The considered downsampling factor varied from 1, corresponding to the actual acquisitions, to 4, corresponding to an average pixel resolution of 16.8 cm^−1^.

### 3.2. Pre-Processing and Linear Regressor

**Figure 1:**
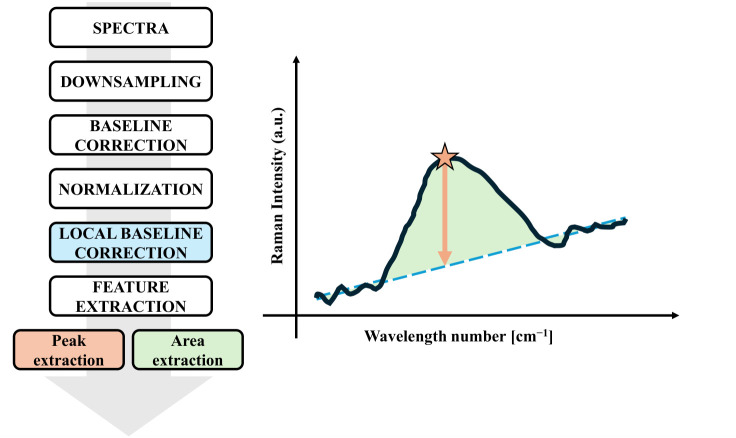
Flowchart of the pre-processing and example of the feature extraction process.

Figure 1 reports the pre-processing protocol.

The typical pre-processing of Raman spectra includes a baseline correction algorithm to remove potential baseline drifts due to fluorescence, a smoothing algorithm to remove high-frequency noise, normalization, and cropping to highlight the specific wavelengths of interest for each analyte. The RamanSpy Framework [13] is employed to make the pre-processing pipeline reproducible. As a baseline removal algorithm, the Asymmetrically Reweighted Penalized Least Squares (ARPLS) is employed on all the wavelengths of the spectra; a smoothing is performed only for the spectra without downsampling with a Savitzy–Golay filter with a window of 5 samples and a polynomial order of 2; for reduced resolutions, no smoothing is performed because the conditions of reduced resolutions are in a way the application of moving average smoothing. This choice was intended to avoid excessive broadening or attenuation of intense Raman bands. The Standard Normal Variate normalization is employed by removing the mean of the spectral values and dividing the amplitude of the spectra by the standard deviation of the Raman intensity.

A crop is performed in the fingerprint region of each analyte. For the nitrate, the spectra are cropped between 1000 and 1080 cm^−1^; for the sulfate, the spectra are cropped between 930 and 1010 cm^−1^; for nitrite, the spectra are cropped between 1240 and 1420 cm^−1^. The following equation is fitted to the experimental points of the cropped region of the Raman spectra S(x):

(1) $$S\left(x\right)=e+dx+a\exp\left(\frac{-{\left(x-b\right)}^{2}}{\left(2c^{2}\right)}\right)$$

where *x* is the wavelength number; the linear baseline e+dx is removed from the experimental data. The peak parameter *a* and the Gaussian area $ac\sqrt{2\pi }$ are used as input features. The peak and area features extracted with the Gauss fitting procedure are called “continuous peak and area”. On the other hand, the discrete peak, computed as the max of the actual experimental data in the considered cropped region, and the discrete area, computed with a trapezoidal method in the cropped region, are also considered as input features.

The single-analyte datasets compose the development set employed to train and validate a linear regressor, which was previously shown to outperform other non-linear machine learning alternatives for the task of concentration regression [6]. The overall performance of the different input features is evaluated on the validation set with a 3-fold cross-validation method. Then, the models are tested in the multi-analyte cases both after and before summation of the spectra.

### 3.3. Performance Metrics

Given a concentration $c_{i}$ for a spectra *i* and a prediction of $\hat{c_{i}}$, the Absolute Error (AE) is computed:

(2) $$AE_{i}=c_{i}-\hat{c_{i}}$$

As a metric of relative performance, the Absolute Percentage Error (APE) is computed:

(3) $$APE_{i}=\frac{c_{i}-\hat{c_{i}}}{c_{i}}$$

The distribution of AE and APE for different input features to the linear regressor and different downsampling factors is reported with boxplots, showing the most suitable feature choice in terms of overall performance and robustness to reduction in spectral resolution.

Then, the Mean Absolute Error (MAE) and Mean Absolute Percentage Error (MAPE) are computed as follows:

(4) $$MAE=\sum_{i=1}^{N}\frac{AE_{i}}{N}$$

(5) $$MAPE=\sum_{i=1}^{N}\frac{APE_{i}}{N}$$

The use of these measures of centrality is common in similar regression studies [6], and these results could be interpreted in terms of overall trends across concentrations, downsampling factors, and employed features. Nevertheless, to better understand the overall effect of the feature choice and downsampling impact of the regression pipeline, a series of statistical tests is also performed. The absolute prediction errors typically appear with a skewed distribution; therefore, non-parametric tests are the most adequate. A Wilcoxon rank test is performed between the area and peak features in the full-resolution spectra scenario to understand if this choice is related to statistically significant differences in prediction error. Thereafter, both features are evaluated across the downsampling factor with a Kruskal–Wallis test and Dunn post hoc analysis with Holm correction [15] to understand whether or not the application of the downsampling statistically affects the performance of the regressor.

Then, as a final analysis, the coefficients of determination ($R^{2}$) are computed for the Gauss fitting procedures across different levels of concentrations and downsampling factors for all the considered analytes to understand whether the Gauss shape assumption remained adequate in spectra with less resolved Raman peaks.

## 4. Results and Discussion

In this section, the results obtained are presented and discussed. Section 4.1 shows the effects of different downsampling factors on the spectral responses of the considered analytes qualitatively. Then, Section 4.2 shows the performance metrics of a linear regression algorithm predicting the concentrations of the three considered analytes with different input features. Similarly, Section 4.3 reports the performance metrics for the same algorithms for predicting the concentration in the mixture-of-analytes case. Section 4.4 explores how the Gauss fitting procedure is altered across concentrations and downsampling factors. Then, Section 5 summarizes the findings and implications found in this study for future research in similar regression-based studies. Finally, Section 6 explores limitations of the proposed methods and possible future directions.

### 4.1. Answer to Q1: Effect of Hardly Discernible Raman Peak Features on the Spectra

In this subsection, the mean spectra of repeated measurements of the three considered analytes are shown in the fingerprint region.

**Figure 2:**
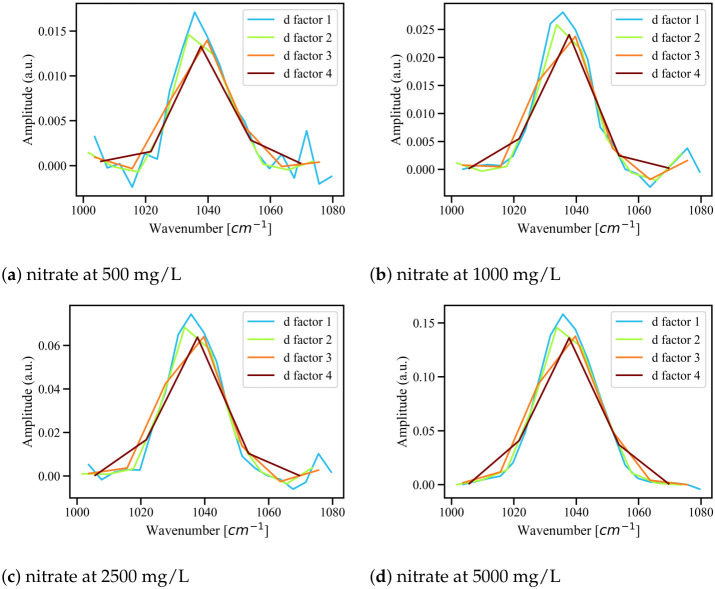
Examples of mean spectra of nitrate at different concentrations and for different downsampling factors d.

Figure 2 presents the mean spectral responses of nitrate solutions at four concentration levels (500, 1000, 2500, 5000 mg/L) computed under different downsampling factors (d). For all concentrations, as expected, the spectra exhibit a well-defined peak in the 1000–1080 cm^−1^ region, which remains consistent across both concentration levels and downsampling conditions.

An increase in nitrate concentration leads to an increase in peak intensity. The overall spectral shape remains similar across concentrations, suggesting that concentration primarily affects signal Raman intensity rather than altering the underlying spectrum shape. Minor variations in peak intensity are observable at lower concentrations, where noise effects are more pronounced. The influence of the downsampling factor is mainly reflected in the smoothness and amplitude of the spectra. Lower values of d retain more intense peak profiles and higher intensity, while larger downsampling factors result in smoother curves with slightly reduced peak heights.

**Figure 3:**
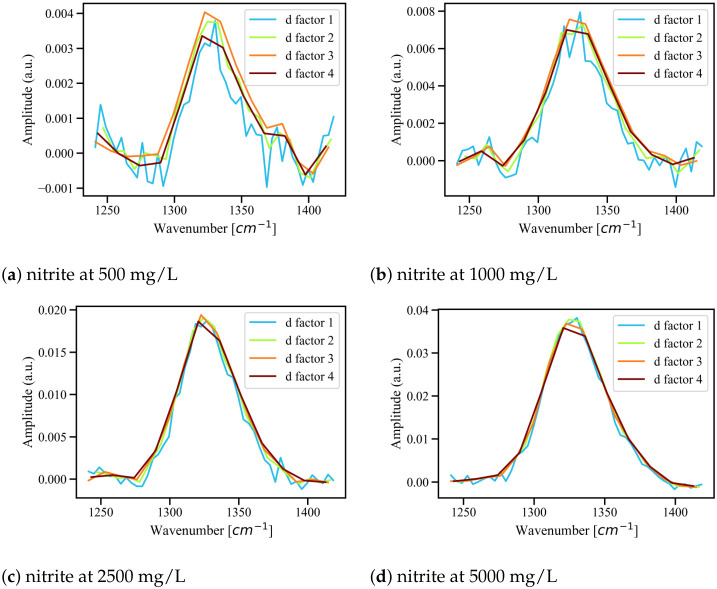
Examples of mean spectra of nitrite at different concentrations and for different downsampling factors d.

Figure 3 illustrates the mean spectra of nitrite at concentrations of 500, 1000, 2500, and 5000 mg/L, obtained using different downsampling factors (d).

In all cases, a characteristic and well-defined peak is observed in the spectral region around 1240–1420 cm^−1^, which is consistent with the known vibrational response of nitrite. As the concentration increases, Raman peak intensity increases, confirming a clear response dependent on concentration. At the lower concentrations (500 and 1000 mg/L), notable deviations between spectra corresponding to different downsampling factors are evident, particularly in the baseline regions and in the vicinity of the main peak. For the full resolution case (d = 1), the spectra exhibit more pronounced noise.

Conversely, at higher concentrations (2500 and 5000 mg/L), the spectra obtained with different downsampling factors show increased smoothness, with minimal distortion of the peak shape or position. Overall, these results demonstrate that while downsampling may influence spectral quality at low nitrite concentrations, its impact becomes less relevant at higher concentrations, where the nitrite spectral signature remains robust and reproducible.

**Figure 4:**
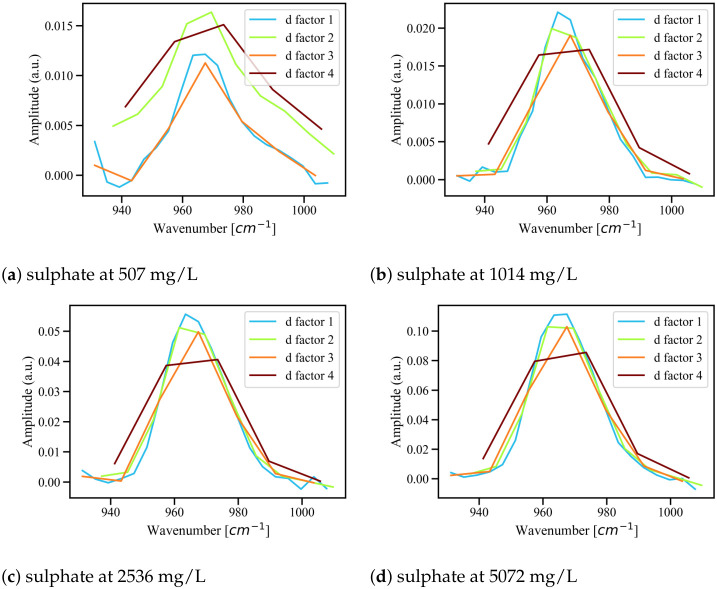
Examples of mean spectra of sulphate at different concentrations and for different downsampling factors d.

Figure 4 shows representative mean spectra of sulphate at concentrations of 507, 1014, 2536, and 5072 mg/L for different downsampling factors (d). In all subfigures, a prominent absorption band is observed in the spectral region around 930–1010 cm^−1^, which is characteristic of sulphate vibrations.

The position of this main peak remains stable across concentrations. As sulphate concentration increases, a clear and progressive increase in peak amplitude is observed, demonstrating a strong response dependent on concentration. At the lowest concentration (507 mg/L), noticeable differences among spectra obtained with different downsampling factors are present, particularly in peak height and baseline behavior. This reflects a greater sensitivity to downsampling under low signal-to-noise conditions, and the failure, to some extent, of the baseline correction algorithm.

At 2536 and 5072 mg/L, the peak shape becomes more defined and more symmetric, and the influence of downsampling on spectral amplitude and shape is markedly reduced. Overall, the figure indicates that downsampling effects are most pronounced at low sulphate concentrations, whereas at higher concentrations, the sulphate Raman peak feature is robust and reproducible, with minimal distortion across different downsampling factors.

### 4.2. Answer to Q2: Single-Analyte Results

This sub-section reports the performance metrics of the three-fold cross-validation of a linear regression model based on four input features. The metrics are aggregated into four concentration ranges based on the quartiles of the distribution for the available concentrations. Firstly, the metrics shown are based on the full-resolution case (d = 1), establishing the best-case scenario and showing the best overall features across different concentration regimes; secondly, the effect of downsampling factors is explored, and the performance is stratified by both features and spectral resolution, responding to the research question Q2.

**Figure 5:**
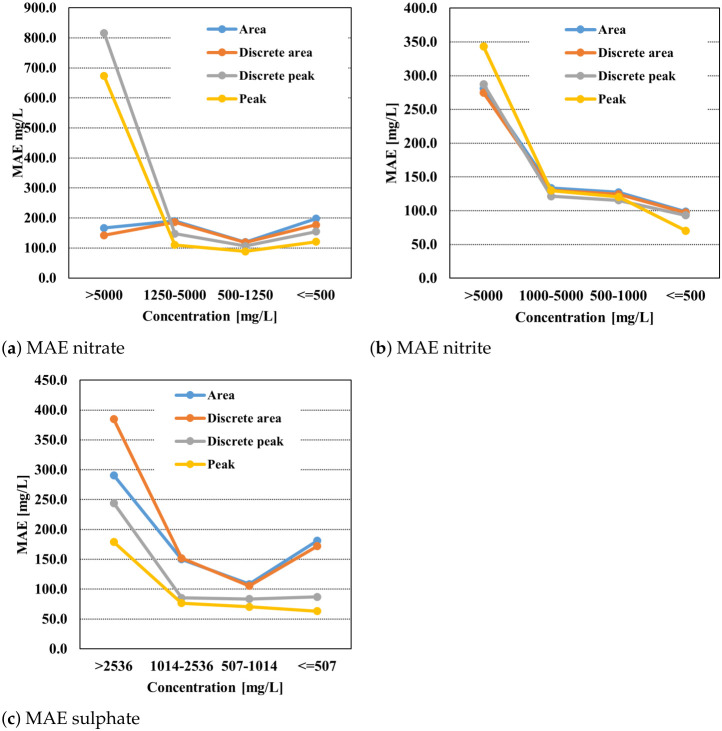
MAE for nitrate (**a**), nitrite (**b**), and sulphate (**c**).

Figure 5 shows the MAE results for the three considered analytes stratified by concentration ranges and extracted features.

As a general trend, the highest MAE results are related to the highest concentration regimes; as the concentration decreases, the MAE approaches lower values.

For sulphate, the peak-based approach yields the best results across all the considered concentration ranges, with the lowest results for the intermediate concentration ranges, with MAE between 70 and 80 mg/L. On the other hand, for nitrate, area-based methods appear to have low and stable prediction errors across the different considered concentration regimes, and the best MAE for the highest concentration range (>5000 mg/L). Nevertheless, peak-based methods remain the best option for intermediate and low concentration regimes, reaching the lowest MAE of 88.3 and 120.5 mg/L for the 1250–5000 mg/L and 500–1250 mg/L ranges. Nitrite displays the smoothest trend among the three species. At very high concentrations (>5000 mg/L), MAE values are elevated for all methods (280 to 350 mg/L), with an advantage for area-based methods over peak-based methods. As concentration decreases, MAE values drop rapidly and consistently, converging in the 1000–5000 mg/L and 500–1000 mg/L ranges. At low concentrations (≤500 mg/L), nitrite prediction has its best performance, with the peak method achieving the lowest MAE of 70.2 mg/L.

**Figure 6:**
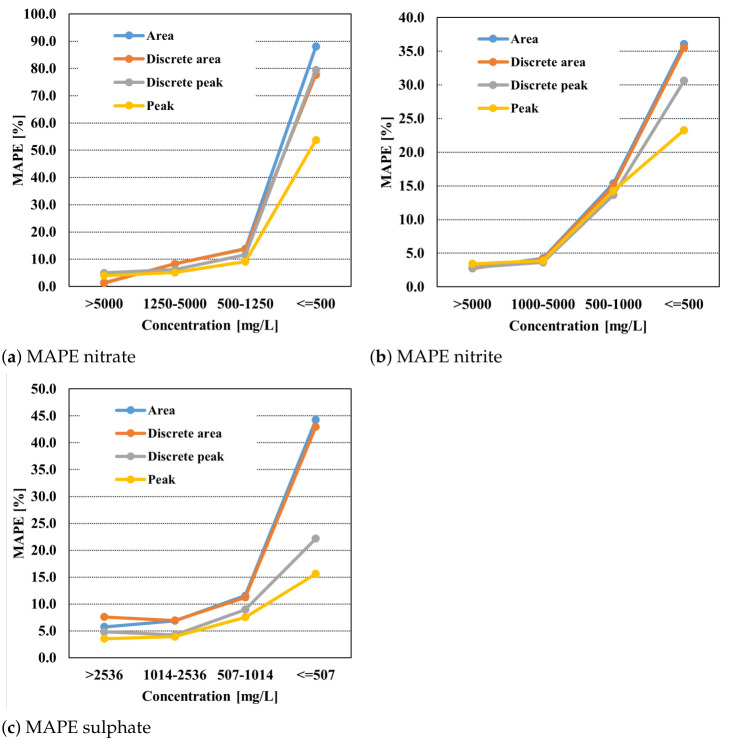
MAPE for nitrate (**a**), nitrite (**b**), and sulphate (**c**).

As another metric, MAPE for the three considered analytes is reported in Figure 6. The results are stratified by concentration ranges and feature-based approaches.

For all analytes, relative errors remain low at the two highest concentration regimes, typically below 9%, regardless of the feature extraction method. This indicates that, when signal amplitude is large, all approaches provide reliable percentage estimates, even in cases where absolute errors may be significant. As concentration decreases, MAPE increases progressively, with the most pronounced errors observed in the lowest concentration range.

Peak-based features consistently yield lower MAPE values at low concentrations across all three analytes. The continuous peak feature provides the best overall relative accuracy, followed by discrete peak representation. This indicates that peak intensity retains a higher percentage sensitivity under weak signal conditions and is less affected by baseline accumulation effects than area-based metrics.

#### Effect of Reduced Resolution on Feature Choice

**Figure 7:**
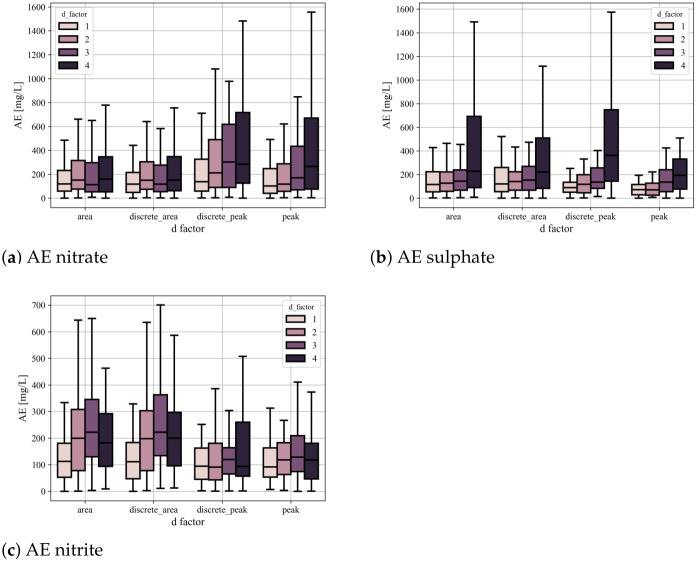
AE for (**a**) nitrate, (**b**) sulphate, and (**c**) nitrite stratified for all d factors in the single-analyte cases.

Figure 7 shows the distribution of AE for linear regression prediction for all three considered analytes stratified by parameters and downsampling factor d.

Across all analytes and all parameters, a consistent trend is observed: AE increases with an increasing downsampling factor. Without any downsampling (d = 1), the median AE is systematically the lowest, and the Inter Quartile Range (IQR) is relatively narrow, indicating both higher performance metrics and lower variability. As d increases from 2 to 4, both the median AE and the IQR increase.

For the peak-based model, the effect of the increase in AE can be explained by peak broadening and attenuation. This is particularly relevant for nitrate and sulphate, which exhibit intense Gaussian-like Raman bands. Conversely, area-based models are less sensitive to local peak-height distortion, but the increase in variability of AE at a lower resolution could be caused by the availability of fewer spectral points for numerical integration and residual baseline errors. Therefore, the widening of the AE IQR with downsampling should be interpreted as a combined effect of peak distortion, reduced sampling density, and baseline-related variability.

This trend is dependent on stronger downsampling, which reduces the effective spectral resolution and degrades peak definition and numerical integration accuracy, propagating into larger concentration errors.

Among the evaluated parameters, area-based metrics exhibit superior robustness to reduced data resolution, achieving low, stable absolute errors and reduced variability across all analytes. Consequently, continuous area integration represents the most reliable feature choice across different downsampling factors, whereas peak-based descriptors can achieve the lowest errors in known conditions of spectra resolution.

### 4.3. Answer to Q2: Results on Mixture of Analytes

**Figure 8:**
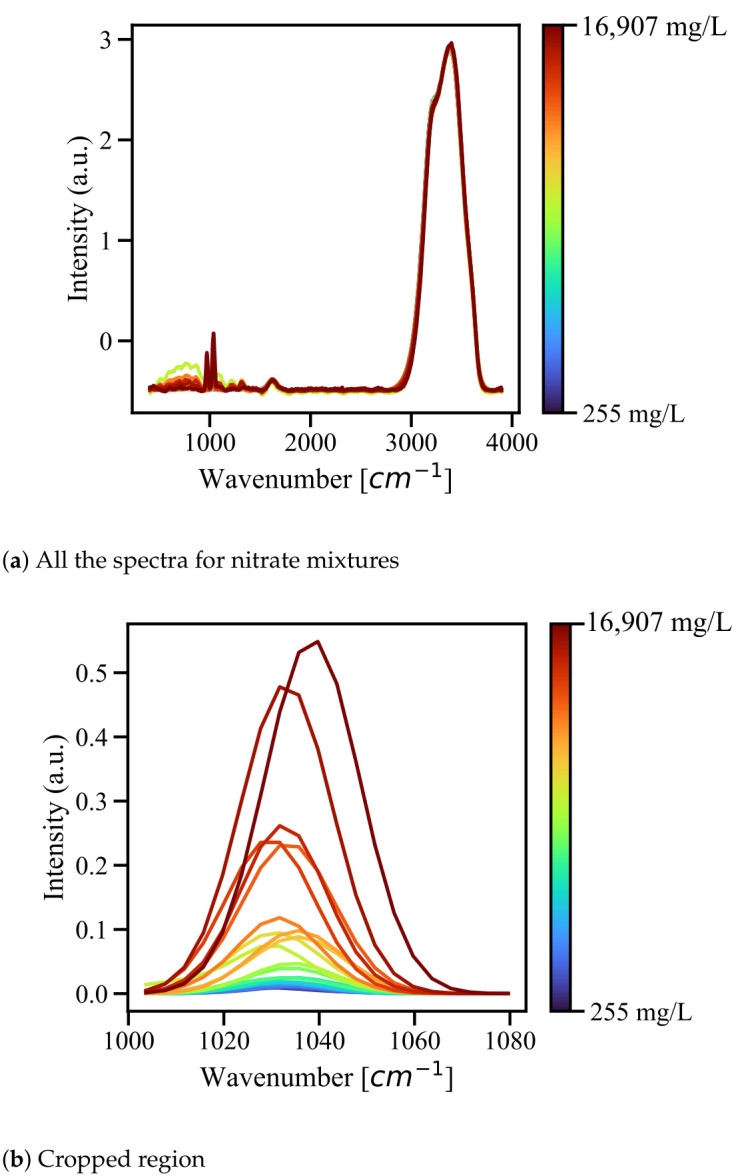
The plot shows the full spectra (**a**) and the cropped spectra (**b**) in mixtures containing nitrate with color-coding related to nitrate concentrations. It is possible to observe a peak shift effect and, to some extent, baseline failures due to the complex dynamic spectra in multi-analyte scenarios.

This sub-section reports the performance of the continuous peak- and area-based linear regressor applied to the mixture of analyte cases. As a way to improve the signal-to-noise ratio of the input spectra, the average spectra across repeated measurements of the same sample are considered. Only concentrations higher than 0 mg/L are included in the analysis. The results are stratified by downsampling factors and feature choice, responding to research question Q2 in a more complex scenario than the previous one. In fact, in conditions of mixtures of analytes, greater wavenumber peak shifts are observed; moreover, the failure of the baseline correction algorithm could potentially hinder the appropriate prediction of the concentration of the analytes. An example is reported in Figure 8.

Even though there are evident peak shifts in the Raman signal, the proposed approach of fitting a Gaussian analytical curve on experimental data is robust to this kind of change, as demonstrated in our previous contribution [6], and the possible mistakes observed with the first baseline correction algorithm are partially solved by the second local correction baseline step.

In the following paragraphs, results are shown in color-coded tables reporting MAE and MAPE, as these measures of centrality are commonly employed in similar work on concentration regression [6]. The color coding depends on the distribution of the considered error, with the green values highlighting the best performances, the yellow values the intermediate errors, and the red values the worst performance. Summing the spectra with the same concentration led to a reduced population size (*n* = 41 for nitrate, *n* = 33 for nitrite, and *n* = 27 for sulphate); a Wilcoxon rank-sum test is performed between peak and area AE and APE for full-resolution spectra to understand whether one or the other method is better; then, a Kruskal–Wallis test is performed to test for possible differences in AE and APE across downsampling factors and selected parameters. The choice of these non-parametric tests is justified by the skewness of the absolute errors.

#### 4.3.1. Nitrate

**Figure 9:**
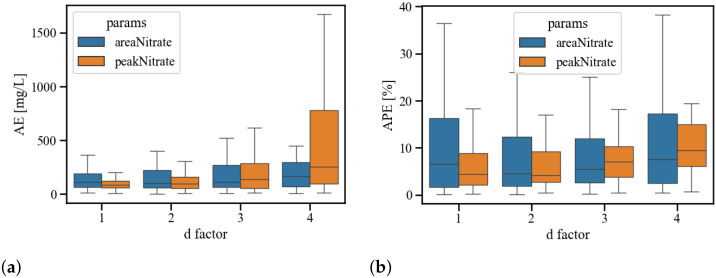
Boxplot for (**a**) AE and (**b**) APE for nitrate for the two selected parameters.

Figure 9 shows the behavior of the prediction errors across downsampling factors for the two selected parameters.

A Wilcoxon rank-sum test was conducted to compare APE and AE between area (n = 41) and peak (n = 41) at full resolution. The median values of AE are 113.77 mg/L and 84.38 mg/L (Z = 1.108, *p* = 0.268) for area and peak, respectively, with no statistically significant difference. The median values of APE are 6.51% and 4.39% for area and peak, respectively, with no statistically significant difference.

A Kruskal–Wallis test was conducted to assess the effect of the downsampling factor on AE and APE. For area-based prediction, no significant differences were observed among the four downsampling conditions (AE distributions show, p=0.660, $\varepsilon^{2}\approx 0.000$; APE distributions, p=0.683, $\varepsilon^{2}=0.000$), and Dunn’s post hoc tests did not reveal any significant pairwise comparisons after Holm correction (all adjusted p=1.000). Conversely, a significant effect of the downsampling factor was detected for the peak-based AE (p=0.001, $\varepsilon^{2}=0.080$). Dunn’s post hoc analysis showed that the prediction-error distribution for d=4 differed significantly from those for d=1 (p=0.001) and d=2 (p=0.011). Inspection of the group medians indicated that d=4 was associated with higher prediction errors than d=1 and d=2. No significant differences were observed among the remaining downsampling conditions after Holm correction. Similarly, a significant effect of the downsampling factor was detected for peak-based APE (p=0.001, $\varepsilon^{2}=0.080$). Dunn’s post hoc analysis showed that the prediction-error distribution for d=4 differed significantly from those for d=1 (p=0.003) and d=2 (p=0.003). Inspection of the group medians indicated that d=4 was associated with higher prediction errors than d=1 and d=2. No significant differences were observed among the remaining downsampling conditions after Holm correction. These findings indicate that peak-based predictions are sensitive to aggressive downsampling, whereas area-based predictions remain comparatively stable across the investigated downsampling levels.

Table 2 and Table 3 summarize the MAE results of the linear regression model for nitrate concentration prediction using area and peak features, evaluated across concentration categories and spectral downsampling factors (d from 1 to 4).

Table 4 and Table 5 summarize the MAPE results of the linear regression model for nitrate concentration prediction using area and peak features, evaluated across concentration categories and spectral downsampling factors (d from 1 to 4).

Across all configurations, prediction accuracy strongly depends on nitrate concentration. Area and peak features achieve their best performance at high nitrate concentrations (c > 3833), with MAPE values consistently below 5% for most downsampling factors. Errors increase progressively toward lower concentration categories, reaching MAPE values close to or above 20% for c ≤ 767. This pattern reflects the reduced signal-to-noise ratio at low nitrate concentrations, where spectral responses become increasingly subtle relative to background noise.

A reduction in spectral resolution from d equal to 1 (4.2 cm^−1^) to d equal to 3 (12.6 cm^−1^) affects both peak- and area-based models slightly; the peak-based model achieves better outcomes in all concentration regimes for d equal to 1 and 2; for d equal to 3, the area-based model has better performance for concentrations higher than 3833 mg/L and concentrations lower than 767 mg/L; a downsampling equal to 4 causes a large decline in performance for both feature strategies, with the peak-based model being the most affected. For example, peak-based MAPE at c ≤ 767 rises from 19.5% (d = 1) to 54.7% (d = 4), accompanied by a corresponding MAE increase from 81.7 to 257.9 mg/L. This trend is consistent with the performed statistical analysis on the overall distribution, which showed the peak-based regression is more sensitive to peak degradation due to downsampling compared to area-based regression.

#### 4.3.2. Nitrite

**Figure 10:**
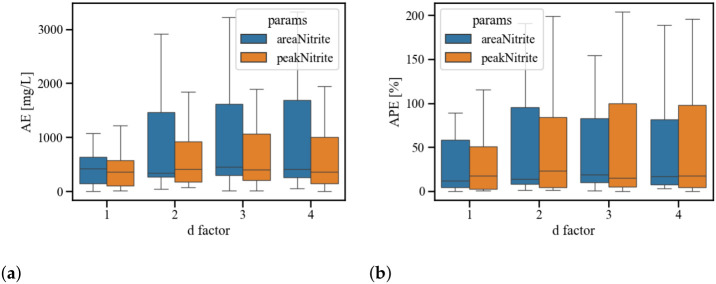
Boxplot for (**a**) AE and (**b**) APE for nitrate for the two selected parameters.

Figure 10 shows the behavior of the prediction errors across downsampling factors for the two selected parameters.

A Wilcoxon rank-sum test was conducted to compare APE and AE between area (n = 33) and peak (n = 33) at full resolution. The median values of AE are 416.69 mg/L and 362.06 mg/L (Z = 0.301, *p* = 0.763) for area and peak, respectively, with no statistically significant difference. The median values of APE are 12.01% and 17.26% for area and peak, respectively, with no statistically significant difference (Z = 0.250, *p* = 0.803).

A Kruskal–Wallis test was conducted to assess the effect of the downsampling factor on APE and AE. However, no significant differences were observed for both prediction errors across downsampling factors given the sample size. For this reason, the tests were extended to the predictions before summation of spectra, where the sample size was deemed to be more appropriate for statistical relevance (n = 330). With regard to AE, the following results are obtained with a Kruskal–Wallis test. For area-based predictions, a significant effect of the downsampling factor was observed (H(3)=32.47, p<0.001, $\varepsilon^{2}=0.022$). Dunn’s post hoc tests with Holm correction revealed that d=1 yielded significantly lower prediction errors than d=2 ($p=1.18\times 10^{-4}$), d=3 ($p=1.1\times 10^{-5}$), and d=4 ($p=5.0\times 10^{-6}$), whereas no significant differences were detected among d=2, d=3, and d=4. Similarly, a significant effect of the downsampling factor was found for the peak-based predictions (H(3)=12.27, p=0.007, $\varepsilon^{2}=0.007$). Post hoc analysis indicated that d=1 produced significantly lower prediction errors than d=2 (p=0.011), d=3 (p=0.023), and d=4 (p=0.050), while no significant differences were observed among the remaining downsampling conditions. Overall, the effect of downsampling was more pronounced for area-based predictions, whereas the effect observed for peak-based prediction was statistically significant but of negligible practical magnitude. This trend is opposite when compared to the one observed in nitrate predictions and could be explained by the characteristic spectral response of nitrite. As the nitrite spectral response is broader and less intense than nitrate, the evaluated fingerprint for nitrite is larger than the one of nitrate, and the estimated peak could be less affected by the downsampling smoothing effect. Concerning the APE, a Kruskal–Wallis test applied to the predictions before summation of the spectra gave the following results. For area-based predictions, a significant effect of the downsampling factor was observed (H(3)=11.99, p=0.007, $\varepsilon^{2}=0.007$). Dunn’s post hoc tests with Holm correction showed that d=1 yielded significantly lower prediction errors than d=3 (p=0.016) and d=4 (p=0.016), whereas the comparison between d=1 and d=2 did not reach statistical significance after correction (p=0.086). No significant differences were detected among d=2, d=3, and d=4. Conversely, no significant effect of the downsampling factor was found for peak-based prediction (H(3)=4.74, p=0.191, $\varepsilon^{2}=0.001$), and Dunn’s post hoc tests did not reveal any significant pairwise comparisons after Holm correction (all adjusted p≥0.365). These findings indicate that downsampling has a statistically detectable but negligible practical effect on area-based prediction errors, while peak-based predictions appear comparatively insensitive to the investigated downsampling levels. Overall, the results show that area descriptors for nitrite predictions are slightly less reliable in conditions of low-quality spectra.

Table 6 and Table 7 summarize the MAE results of the linear regression model for nitrite concentration prediction using area and peak features, evaluated across concentration categories and spectral downsampling factors (d from 1 to 4).

Table 8 and Table 9 summarize the MAPE results of the linear regression model for nitrite concentration prediction using area and peak features, evaluated across concentration categories and spectral downsampling factors (d from 1 to 4).

Overall, the results reveal substantially higher prediction errors for nitrite compared with nitrate, with pronounced sensitivity to both concentration level and spectral resolution. In contrast to the trends for nitrate prediction, the area-based model in the nitrite scenario has good results for the full-resolution case, which decline rapidly with the reduction in spectral resolution. On the other hand, the peak-based model shows, as before, a better overall outcome compared to the area-based model and also good stability in prediction error across the reduction in spectral resolution for concentrations higher than 1916 mg/L. This inversion of the trend for peak and area is also consistent with the previous statistical tests. Nitrite peak appears to be a more reliable feature than area descriptors, probably due to the broader spectral response of the nitrite and its weak signal intensity.

The prediction accuracy strongly degrades with decreasing nitrite concentration for both feature types and all downsampling factors. At high concentrations (c > 3833), models achieve low percentage errors, with MAPE values below 10% for area-based features and below 4% for peak-based features across all d. In contrast, for the lowest concentration category (c ≤ 767), MAPE values increase dramatically, exceeding 150% for area-based models and reaching nearly 200% for peak-based models. This sharp deterioration reflects the extremely weak spectral signal associated with low nitrite concentrations, where instrumental noise and background effects dominate the response. The corresponding MAE values confirm this trend, increasing from a few hundred mg/L at high concentrations to well above 700–900 mg/L at low concentrations.

Peak-based features outperform area-based features at high nitrite concentrations, exhibiting lower MAPE and MAE values for concentrations higher than 1916. This suggests that, when the nitrite signal is strong, spectral peaks provide informative and well-localized predictors. For the concentrations below 1916 mg/L, neither the peak nor the area performs well.

At high concentrations, peak-based MAPE remains relatively stable across d, changing from 1.9% to 3.3%, whereas area-based MAE increases steadily from 2.9 to 8.6%. At intermediate and low concentrations, both MAPE and MAE worsen rapidly with increasing d, especially for the 767–1916 band, where area-based MAE more than quadruples from d equal to 1 to d equal to 4.

In contrast to nitrate, nitrite prediction using linear regression appears fundamentally constrained by signal detectability rather than model configuration. High prediction errors at low concentrations, combined with strong sensitivity to spectral resolution, are coherent with the weak and broad nitrite spectral responses that are poorly captured by linear models under realistic noise conditions. The peak feature is preferable at high concentrations (c > 1916 mg/L) and seems to maintain a reliable prediction with an increase in downsampling.

#### 4.3.3. Sulphate

**Figure 11:**
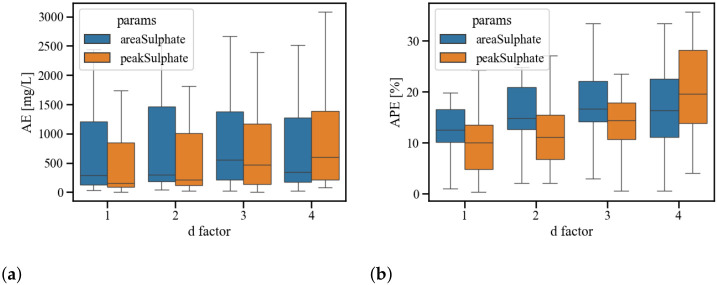
Boxplot for (**a**) AE and (**b**) APE for sulphate for the two selected parameters.

Figure 11 shows the behavior of the prediction errors across downsampling factors for the two selected parameters.

A Wilcoxon rank-sum test was conducted to compare APE and AE between area (n = 27) and peak (n = 27) at full resolution. The overall median values of AE are 292.76 mg/L and 153.76 mg/L for area and peak, respectively, with no statistically significant difference (Z = 1.064, *p* = 0.287). The overall median values of APE are 12.58% and 10.02 % for area and peak, with no statistically significant difference (Z = 1.808, *p* = 0.071). A Kruskal–Wallis test was conducted to assess the effect of the downsampling factor on AE. For peak-based predictions, no significant differences were observed among the four downsampling conditions (H(3)=1.04, p=0.793, $\varepsilon^{2}=0.000$), and Dunn’s post hoc tests did not reveal any significant pairwise comparisons after Holm correction (all adjusted p=1.000). Similarly, no significant effect of the downsampling factor was detected for peak-based regression (H(3)=5.00, p=0.172, $\varepsilon^{2}=0.019$), and no Dunn–Holm pairwise comparison reached statistical significance (all adjusted p≥0.206). Although median prediction errors for peak-based predictions increased numerically from d=1 to d=4, as can be seen in Figure 11, the rank-based analysis did not provide sufficient evidence for statistically significant distributional differences across downsampling factors. Kruskal–Wallis tests were conducted to assess the effect of the downsampling factor on sulphate prediction errors, considering both absolute error (AE) and absolute percentage error (APE). For AE, no significant differences were found across the four downsampling conditions for either area-based predictions (H(3)=1.04, p=0.793, $\varepsilon^{2}=0.000$) or peak-based predictions (H(3)=5.00, p=0.172, $\varepsilon^{2}=0.019$); accordingly, Dunn’s post hoc tests with Holm correction did not identify any significant pairwise comparisons (area-based: all adjusted p=1.000; peak-based: all adjusted p≥0.206). Although median prediction errors for peak-based predictions increased numerically from d=1 to d=4, the rank-based analysis did not provide sufficient evidence for statistically significant distributional differences across downsampling factors. For APE, no significant effect of downsampling was observed for area-based predictions (H(3)=4.35, p=0.226, $\varepsilon^{2}=0.013$), with no significant Dunn–Holm pairwise differences (all adjusted p≥0.257). In contrast, peak-based predictions showed a significant effect of downsampling on APE (H(3)=14.29, p=0.003, $\varepsilon^{2}=0.109$). Dunn’s post hoc analysis indicated that the prediction-error distribution for d=4 differed significantly from those for d=1 (p=0.003) and d=2 (p=0.019). Inspection of the group medians indicated that d=4 was associated with higher percentage errors than the lower downsampling conditions. Overall, these findings suggest that area-based sulphate predictions were more robust to downsampling, whereas peak-based predictions were more sensitive to downsampling, particularly when the prediction error was expressed in relative terms using APE.

Table 10 and Table 11 summarize the MAE results of the linear regression model for sulphate concentration prediction using area and peak features, evaluated across concentration categories and spectral downsampling factors (d from 1 to 4).

Table 12 and Table 13 summarize the MAPE results of the linear regression model for sulphate concentration prediction using area and peak features, evaluated across concentration categories and spectral downsampling factors (d from 1 to 4).

In contrast to nitrate and nitrite, sulphate predictions seem to have comparatively weaker sensitivity at higher concentrations.The improvements are due to the presence of outliers influencing the mean prediction error. When observing the median AE and APE for c < 767 mg/L in Table 14, it is possible to find the expected trend of increased error as the downsample increases.

Similarly to nitrate, the peak-based model outperforms the area-based model for all downsampling factors except d equal to 4; the peak-based model exhibits a slight decline in performance as the spectral resolution reduces, whereas the area-based model maintains a stable prediction error across different spectral resolutions. This trend is consistent with the statistical analysis, where the distributions of area-based predictions were not statistically different for both AE and APE. This trend is similar to the one observed for nitrate and probably depends on the similar intense Gaussian shape of nitrate and sulphate, which benefits from the peak feature if the resolution is high enough.

Prediction accuracy is strongly dependent on sulphate concentration. At high and intermediate concentrations (c > 1916), both feature types yield relatively low and stable errors, with MAPE values generally between 7 and 15%. In these regimes, sulphate prediction appears robust across downsampling levels, indicating that the dominant spectral information is preserved even under reduced spectral resolution. The best performance is achieved with the peak-based model without downsampling, with MAPE of 7.2 % for concentrations above 7665 mg/L and MAPE of 8.0% for concentrations between 1916 and 7665 mg/L. At the lowest concentration band (c ≤ 767), errors increase dramatically at full spectral resolution (d = 1), particularly for area-based features, with MAPE close to 764%, and MAE close to 3169 mg/L. This highlights severe instability in sulphate prediction at low concentrations, likely due to noise and weak signal strength. However, unlike nitrate and nitrite, sulphate exhibits a pronounced recovery in performance as spectral downsampling increases.

Increasing d leads to reductions in both MAPE and MAE for c ≤ 767 for both feature types. For area-based models, MAPE decreases from 764% (d = 1) to 143% (d = 4), with a corresponding MAE reduction from 3169 to 655 mg/L. A similar trend is observed for peak-based features, where MAE decreases from 1289 to 655 mg/L and MAPE from approximately 303% to 129%.

At intermediate concentrations between 767 and 1916 mg/L, downsampling does not seem to affect the performance of the area-based model, whereas it generally worsens the performance of the peak feature. At high concentrations (c > 7665), downsampling induces a slight degradation in MAE for both feature types, particularly at d equal to 3 or 4. The decline in performance is more accentuated for the peak-based model rather than the area-based model.

Peak-based features consistently outperform area-based features at high and intermediate concentrations, yielding lower MAPE and MAE values across all downsampling factors. For downsampling factors from 1 to 3 and concentrations above 1916 mg/L, MAPE for the peak-based model is within 15%. This indicates that, when sulphate concentrations are sufficiently high, localized peak information provides an efficient and accurate representation of the spectral signal. At low concentrations, however, both feature types exhibit severe degradation at full resolution, and their performance converges as downsampling increases. Area-based features show larger improvements with increasing d, reflecting the robustness of this feature across downsampling factors.

### 4.4. Effect of Downsampling on Pre-Processing Pipeline and Gaussian Fitting Procedure

**Figure 12:**
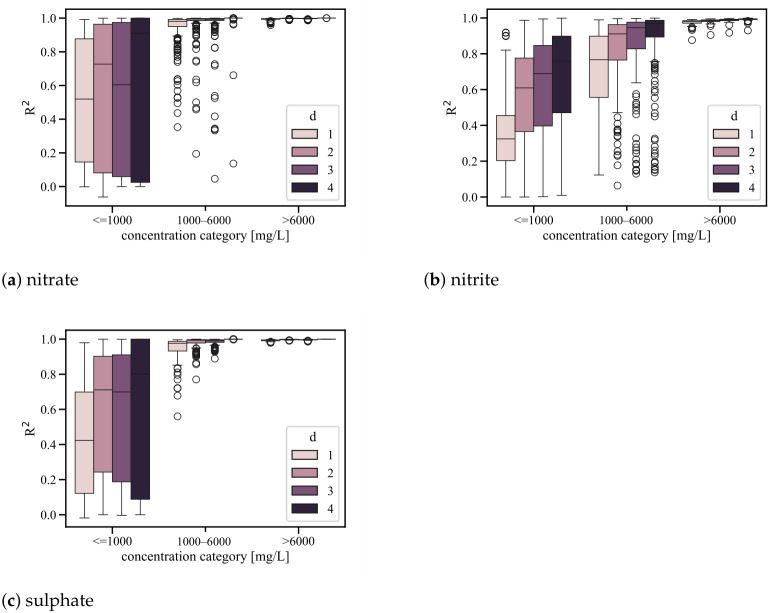
Distribution of the Gaussian fitting coefficient of determination ($R^{2}$) of Equation (1) for nitrate (**a**), nitrite (**b**), and sulphate (**c**), stratified by concentration category and downsampling factor d.

To verify whether the degradation observed at higher downsampling factors originated from a failure of the Gaussian model, the goodness-of-fit of Equation (1) was evaluated across all analytes, concentration ranges, and downsampling factors. Figure 12 reports the distribution of the coefficient of determination ($R^{2}$) of the Gaussian model of Equation (1) for nitrate, nitrite, and sulphate as a function of concentration category and downsampling factor.

For all three analytes, the quality of the Gaussian fit is strongly concentration dependent. At high concentrations (>6000 mg/L), the fitted peaks are almost perfectly described by the Gaussian model, with median $R^{2}$ values close to 1.0 and very limited variability regardless of the downsampling factor. This indicates that the reduction in spectral resolution up to d = 4 does not substantially alter the overall Gaussian behavior of the Raman bands when the signal-to-noise ratio is high. In the intermediate concentration range (1000–6000 mg/L), the Gaussian approximation remains highly accurate for all analytes and all downsampling factors. Although a larger number of low $R^{2}$ outliers is observed, particularly for nitrite and sulphate, the central tendency remains close to unity, suggesting that most spectra are still well represented by a Gaussian peak even after resolution degradation. It is possible to notice that the nitrite for this concentration range has the lowest median values. The largest variability is observed at concentrations below 1000 mg/L. In this regime, the distributions become broader and median $R^{2}$ values decrease substantially, reflecting the increasing influence of noise and baseline fluctuations on the fitting process. Interestingly, for nitrate and nitrite, the median $R^{2}$ tends to increase as the downsampling factor increases, indicating that moderate spectral averaging suppresses high-frequency noise and improves the stability of the Gaussian fit. Sulphate exhibits a similar tendency, although with a larger spread of values.

The analysis showed that median $R^{2}$ values remained close to unity for medium and high concentrations even at d = 4, indicating that the Gaussian model continues to accurately represent the broadened Raman peaks. Consequently, the reduction in regression performance previously observed at low spectral resolution is more likely related to the loss of peak information and reduced feature sensitivity rather than to an inadequate Gaussian fit.

## 5. Implications for Raman Feature Selection in Regression-Based Studies

In this section, a summary of the findings is reported with practical implications for Raman feature selection in regression-based studies. For intense Gaussian-line responses as in the case of nitrate and sulphate, peak-based regression is highly affected by a reduction in the quality of the spectra; on the other hand, area-based regression exhibited a robust behavior showing stable prediction errors across the tested conditions. Nitrite has a different spectral response, characterized by a broader Gaussian-line shape. The observed trend was the opposite. Peak-based regression obtained the best and most stable results across the tested conditions, whereas area-based regression was affected by the loss of quality in the spectra. As an implication, broader and weaker responses appear to be more effectively captured by the extracted peak, whereas the more intense responses appear to be more effectively described by the area.

## 6. Limitations and Future Directions

In this study, linear regression was adopted because the pre-processing pipeline and feature-extraction stage already provide good descriptors of the concentration levels. After baseline correction, normalization, and extraction of peak or area-based descriptors, a simple regression model provides an interpretable way to evaluate the effect of spectral resolution on quantification performance. As a consequence, the effect of hardly discernible Raman peak features cannot be extended to any regressor but is limited to the employed choice of linear regression. Concerning the pre-processing stage, the assumption of a linear local baseline could fail in complex real matrices, leading to a propagation of the error throughout the entire pre-processing pipeline. More complex non-linear models may be beneficial in alternative frameworks where raw or minimally processed spectra are used as input. In particular, neural network-based models could potentially learn relevant spectral features, baseline effects, and non-linear relationships directly from the spectra, reducing reliance on handcrafted pre-processing. This approach may be especially relevant for weak-signal analytes such as nitrite at low concentrations, although it would require larger datasets and dedicated validation, and could be a future line of research. Additionally, Savitzky–Golay smoothing was applied only to the full-resolution spectra (d = 1), whereas the downsampling procedure generated different sets of spectra through a non-overlapping sliding-window averaging procedure that inherently provides a smoothing effect by averaging adjacent spectral points. Additional smoothing was therefore not applied in order to avoid excessive broadening of Raman bands and alterations in peak intensity, particularly for narrow spectral features such as nitrate and sulphate peaks. Although downsampling resulted in smoother spectra and reduced peak intensity, the potential benefit of further smoothing for low-concentration samples, where noise and residual baseline fluctuations are more dominant, cannot be excluded. However, the objective of the present study was to isolate the effect of spectral-resolution reduction while maintaining a consistent pre-processing workflow across all resolution levels. Introducing an additional smoothing step would add a further tunable denoising parameter, making it more difficult to distinguish the individual contributions of the downsampling factor and noise filtering to model performance. Therefore, a systematic optimization of smoothing parameters for downsampled spectra was deemed to be beyond the scope of the current work and is identified as an important direction for future research, particularly through adaptive smoothing strategies tailored to spectral resolution and the signal-to-noise ratio. With regard to noise management and peak sensitivity, a limitation of the present approach is its reliance on conventional Raman spectroscopy, which remains susceptible to background noise and fluorescence interference that can obscure weak chemical signals. Emerging enhancement strategies, such as spread spectrum surface-enhanced Raman spectroscopy, have demonstrated the ability to suppress unrelated noise through pseudorandom temporal encoding and autocorrelation-based signal reconstruction, resulting in substantial signal-to-noise ratio improvements and markedly enhanced detection sensitivity [16]. Although spread spectrum surface-enhanced Raman spectroscopy was beyond the scope of this study, the integration of similar noise-reduction and signal-enhancement methodologies could further improve the sensitivity and detection limits of the proposed Raman-based framework in future investigations.

## 7. Conclusions

This study provides a comprehensive analysis of the effects of poorly resolved Raman peaks in the quantification of inorganic pollutants by Raman spectroscopy using feature-based machine learning. Downsampling the Raman signal leads to smoother spectra and reduced peak intensity, which in turn negatively affects concentration prediction error. The impact is particularly pronounced at low analyte concentrations, where signal-to-noise ratio limitations amplify the effects of loss of peak intensity. A dataset consisting of the Raman spectra of nitrate, nitrite, and sulphate is employed for the training and testing of a concentration regressor. The pre-processing pipeline is composed of baseline correction, normalization, smoothing, local baseline removal, and Raman feature extraction. A linear regressor is trained on peak and area features extracted in the training set composed of the spectra with the single analyte. The tests are conducted in the scenario with a mixture of analytes. To simulate the worsening of spectra quality, a downsampling of the Raman signal is applied, and the previous procedure is repeated across the tested downsampling factors from 1 to 4, testing in which way the extracted features behaved to smoother and less resolved Raman signals. Peak-based regression for nitrate and sulphate was the most affected by the decrease in feature quality across the downsampling factors. In particular, a significant effect for the peak-based absolute prediction errors and absolute percentage prediction errors was detected for nitrate (*p* = 0.001, $\epsilon^{2}$ = 0.080) and a significant effect for the absolute percentage prediction error was detected for sulphate (*p* = 0.003, $\epsilon^{2}$ = 0.109). On the other hand, area-based predictions were not affected statistically in the analyzed error distributions across the downsampling factors, demonstrating a higher robustness to the loss of quality in the Raman spectra. The best results for nitrate for area-based regression are obtained for concentrations higher than 3833 mg/L with MAPE under 7%. In the medium concentration range between 1916 mg/L and 3833 mg/L, area-based regression obtained a MAPE under 16% for all tested conditions. The best results for sulphate are obtained after 1916 mg/L, where area-based regression obtains results mostly within a MAPE of 16%. The results obtained for nitrite have an opposite trend justified by the broader shape of nitrite. Area-based regression appears more sensitive to signal downsampling than peak-based regression. In particular, a statistically significant difference in absolute prediction-error distribution (*p* < 0.001, $\epsilon^{2}$ = 0.022) and absolute percentage error distribution (*p* = 0.007, $\epsilon^{2}$ = 0.007) was found for area-based regression across downsampling factors. On the other hand, peak-based regression was a more reliable approach in this case, with no statistically significant difference across downsampling factors. The best results for nitrite are obtained for concentrations higher than 3833 mg/L with a peak-based regression method and a MAPE under 4%; in the medium range of concentrations between 1916 mg/L and 3833 mg/L, the MAPE is always under 19% for all tested conditions.

## Figures and Tables

**Table 1:** Dataset description.

Analytes	Concentrations [mg/L]	Total Examples
${\mathrm{NO}}_{3}^{-}$	122; 250; 306; 500; 613; 800; 1000; 1250; 1533; 2500; 3066; 5000; 6133; 15,333; 30,677	149
${\mathrm{SO}}_{4}^{2-}$	253; 507; 845; 1014; 1692; 2536; 5072	70
${\mathrm{NO}}_{2}^{-}$	250; 500; 750; 1000; 2500; 5000; 10,000	70
${\mathrm{NO}}_{3}^{-}{\mathrm{/SO}}_{4}^{2-}$	(422/422); (676/3381), (845/845); (1690/8453); (1690/1690); (3381/16,907); (3381/3381); (16,907/16,907)	75
${\mathrm{NO}}_{3}^{-}{\mathrm{/NO}}_{2}^{-}$	(383/1916); (511/511); (767/3833); (1022/1022); (1533/7666); (1533/1533); (1916/383); (3066/3066); (3067/15,333); (3833/767); (7655/7655); (7666/1533); (15,333/15,333); (15,333/3067)	140
${\mathrm{NO}}_{3}^{-}{\mathrm{/NO}}_{2}^{-}{\mathrm{/SO}}_{4}^{2-}$	(255/255/255); (383/1916/383); (383/383/1916); (511/511/511); (767/3833/767); (767/767/3833); (1022/1022/1022); (1533/1533/1533); (1533/7666/1533); (1533/1533/7666); (1916/383/383); (3066/3066/3066); (3067/15,333/3067); (3067/3067/15,333); (3833/767/767); (7665/7665/7665); (7666/1533/1533); (15,333/15,333/15,333); (15,333/3067/3067)	190
All spectra	-	694

**Table 2:** LR nitrate area MAE [mg/L].

d	c > 3833	3833 ≥ c > 1690	1690 ≥ c > 767	c ≤ 767
**1**	248.4	238.9	114.3	106.8
**2**	329.4	302.5	108.6	79.4
**3**	385.3	305.0	106.7	78.6
**4**	573.9	438.5	121.3	140.2

**Table 3:** LR peak nitrate MAE [mg/L].

d	c > 3833	3833 ≥ c > 1690	1690 ≥ c > 767	c ≤ 767
**1**	263.9	124.2	69.3	81.7
**2**	281.0	147.6	69.0	76.3
**3**	624.2	194.4	86.2	100.2
**4**	1279.4	453.6	131.2	257.9

**Table 4:** LR area nitrate MAPE [%].

d	c > 3833	3833 ≥ c > 1690	1690 ≥ c > 767	c ≤ 767
**1**	2.2	8.7	8.8	23.7
**2**	3.3	11.0	7.8	18.1
**3**	3.8	11.0	7.8	17.2
**4**	6.3	15.9	8.6	29.7

**Table 5:** LR peak nitrate MAPE [%].

d	c > 3833	3833 ≥ c > 1690	1690 ≥ c > 767	c ≤ 767
**1**	2.6	5.0	5.4	19.5
**2**	2.3	5.5	5.1	17.9
**3**	5.3	6.6	5.9	21.6
**4**	11.7	15.7	9.0	54.7

**Table 6:** LR area nitrite MAE [mg/L].

d	c > 3833	3833 ≥ c > 1916	1916 ≥ c > 767	c ≤ 767
**1**	355.8	373.4	414.2	764.8
**2**	850.0	920.2	1602.3	776.2
**3**	965.4	1022.5	1625.9	773.1
**4**	1043.9	1029.3	2117.4	716.1

**Table 7:** LR peak nitrite MAE [mg/L].

d	c > 3833	3833 ≥ c > 1916	1916 ≥ c > 767	c ≤ 767
**1**	232.1	391.0	537.3	942.5
**2**	376.6	615.2	855.3	842.4
**3**	409.6	597.6	875.7	777.4
**4**	323.9	599.9	1014.5	929.4

**Table 8:** LR area nitrite MAPE [%].

d	c > 3833	3833 ≥ c > 1916	1916 ≥ c > 767	c ≤ 767
**1**	2.9	11.4	29.1	156.7
**2**	7.1	27.1	116.6	157.6
**3**	8.1	30.3	117.4	161.6
**4**	8.6	30.4	149.7	150.6

**Table 9:** LR peak nitrite MAPE [%].

d	c > 3833	3833 ≥ c > 1916	1916 ≥ c > 767	c ≤ 767
**1**	1.9	11.9	37.0	193.2
**2**	3.2	18.2	62.6	176.7
**3**	3.3	17.6	63.9	162.5
**4**	2.6	17.6	73.2	175.3

**Table 10:** LR area sulphate MAE [mg/L].

d	c > 7665	7665 ≥ c > 1916	1916 ≥ c > 767	c ≤ 767
**1**	1465.3	454.9	395.2	3169.3
**2**	1709.8	499.4	417.1	2880.5
**3**	1924.1	648.8	235.3	1603.2
**4**	1572.6	464.5	236.8	664.8

**Table 11:** LR peak sulphate MAE [mg/L].

d	c > 7665	7665 ≥ c > 1916	1916 ≥ c > 767	c ≤ 767
**1**	1003.2	326.4	242.7	1288.8
**2**	1213.2	373.6	244.1	1125.0
**3**	1652.6	462.4	388.1	772.6
**4**	2178.1	596.4	243.1	665.4

**Table 12:** LR area sulphate MAPE [%].

d	c > 7665	7665 ≥ c > 1916	1916 ≥ c > 767	c ≤ 767
**1**	10.7	10.6	26.8	764.1
**2**	12.5	11.8	28.6	677.4
**3**	14.6	16.1	17.5	362.0
**4**	11.4	10.5	16.6	143.3

**Table 13:** LR peak sulphate MAPE [%].

d	c > 7665	7665 ≥ c > 1916	1916 ≥ c > 767	c ≤ 767
**1**	7.2	8.0	16.4	302.6
**2**	8.7	9.3	16.8	257.6
**3**	11.9	11.1	26.9	171.4
**4**	16.0	14.5	17.7	128.8

**Table 14:** Median APE and AE for peak- and area-based sulphate predictions across downsampling factors.

d	Peak Sulphate		Area Sulphate	
	Median APE [%]	Median AE [mg/L]	Median APE [%]	Median AE [mg/L]
1	18.4	77.7	14.7	62.0
2	22.6	114.2	24.8	126.6
3	31.4	132.7	49.7	209.6
4	118.7	602.9	45.5	327.4

## Data Availability

Available upon request.
